# Assimilated Rank of the Civil Branch of the Navy

**Published:** 1848-02

**Authors:** 


### ASSIMILATED RANK OF THE CIVIL BRANCH OF THE NAVY.

We find upon our table a well written tract of some eight pages, of which the above is the text. It seems that a movement has been made in the popular branch of our National Legislature, which may tend to alter or abolish the late regulation by which the officers constituting the civil branch of the navy are assigned definite rank, so as to protect them against the presumption and thoughtless arrogance of their juniors, who happen to belong to the fighting class. How the senior naval officers can countenance measures which must of necessity lessen the respectability of those upon whose skill and integrity much of their comfort and even safety depends, is unaccountable. One would naturally suppose that they would be anxious to surround themselves with the most accomplished men in their several departments, and for that purpose would be the strenuous advocates of measures calculated to induce such to engage in the service. Already, it is well known, much difficulty exists in getting properly qualified physicians to enter the navy, and if measures are adopted which shall subject them to petty annoyances and insults from those beneath them in age, intelligence, and personal character, it is easy to see what the consequence will be :—the best men will resign and seek more honourable employment, and others, who would adorn any branch of the public service, will shun appointments which hold out neither the prospect of wealth nor social respectability.

